# Comparative Effectiveness of Ultrasound-Guided Intratendinous Prolotherapy Injection with Conventional Treatment to Treat Focal Supraspinatus Tendinosis

**DOI:** 10.1155/2018/4384159

**Published:** 2018-07-02

**Authors:** John George, Shyan Ch'ng Li, Zulkarnain Jaafar, Mohamad Shariff A. Hamid

**Affiliations:** ^1^University of Malaya Research Imaging Centre, Faculty of Medicine, University of Malaya, 50603 Kuala Lumpur, Malaysia; ^2^Department of Biomedical Imaging, Faculty of Medicine, University Malaya Medical Center, 50603 Kuala Lumpur, Malaysia; ^3^Department of Sports Medicine, Faculty of Medicine, University of Malaya, 50603 Kuala Lumpur, Malaysia

## Abstract

**Objective:**

To evaluate the efficacy of dextrose prolotherapy injection for focal supraspinatus rotator cuff tendinosis via outcomes such as functional score, range of movement, and real-time ultrasound parameters.

**Materials and Methods:**

12 adult patients with focal supraspinatus tendinosis recruited after they had less than 30% improvement in functional (DASH) scores after one month of physiotherapy following initial presentation. Seven patients had 0.5–1.0 ml of prolotherapy injection (12.5% dextrose, 0.5% lignocaine) injected into the area of focal tendinosis under ultrasound guidance. Meanwhile, five patients continued standard physiotherapy with no intervention performed. Regional area of echogenicity in decibels, DASH, range of movements of the shoulder, pain, and sleep scores were measured at baseline and at 12 weeks.

**Results:**

The prolotherapy group showed significant improvement in shoulder abduction (*p*=0.030) and an improvement in sleep score (*p*=0.027). The echogenicity of area of tendinosis significantly increases at the end of treatment (*p*=0.009). However, there was a nonsignificant reduction in pain score in the injection group (43.5%) and in the control group (25%) at 12 weeks (*p* > 0.005).

**Conclusion:**

Ultrasound-guided intratendinous prolotherapy injection significantly improves patient's range of abduction and improves sleep within 12 weeks of treatment compared to conventional physiotherapy management.

**Trial Registration:**

This study was registered under Current Controlled Trials (UK) and given International Standard Randomised Controlled Trial Number (ISRCTN) of 43520960.

## 1. Introduction

Shoulder pain is an important condition of the upper extremity occurring in approximately 15 in 1000 patients per year in the outpatient primary care setting and affects one in three individuals during their lifetime [1]. There were almost 7.5 million visits to the doctors in 2006 due to shoulder pain, and 8–13% involved athletic injuries [2]. Normal range of shoulder movement is needed for daily movement, and restrictions to the activities of daily living are remarkable when someone is experiencing shoulder pain. Rotator cuff tendinopathy (RCT) is a main cause of shoulder pain and disability [1] and occurs in about one in five symptomatic shoulders. It is commonly seen in manual workers, athletes, and elderly; shoulder pain and weakness will interfere with their work, sleep, self-care, physical activities and sports [3]. Painful tendinopathy is challenging to treat [4] and the nonoperative conservative treatment is the first-line treatment for most RCT. The conservative treatment approaches frequently involving rest, activity modification, physical therapy, and pain medication [1].

Tendinopathy is a common painful condition with reduced functional capacity of the tendon associated with the histopathological findings showing failed healing response [4]. Moreover, little is known about the pathogenesis of tendon pain in the early stages and frequently asymptomatic but the tendinopathic changes in tendon are progressive. However, many patients can be symptomatic when there is a temporary increase in tendon loading, but the symptoms could settle spontaneously and recur at later time, producing a cyclical pattern of symptoms and remission [5]. The Institute of Medicine describes chronic pain as pain that persists for a period of three to six months or beyond the time of normal healing [6]. Tendinopathy is a difficult problem requiring quite comprehensive management, and patients often respond poorly to treatment [7]. Tendinopathy shows features of disordered healing and inflammation that are not typically seen, and preexisting degeneration has been implicated as a risk factor for acute tendon rupture [7, 8]. It has been identified that natural healing of tendon is slow and most often may not heal back to their original strength or endurance as tendon tissues have poor blood supply [9].

Even though extensive studies using anti-inflammatory drugs in the management of RCT have been done and pain relief has been achieved in many controlled studies, investigation on tendon healing was not performed in these studies [10]. Moreover, if pain has not improved after a prescribed time of anti-inflammatory drugs and physiotherapy, which varies from practice to practice, the patient's pain and functional scores are re-evaluated. If there is little improvement in pain and functional scores, a subacromial corticosteroid injection could be provided and physiotherapy could be continued. The response to steroid injection is quite variable and often patients complain of symptoms of tendinosis for many months [11]. Steroid injections' role is to reduce inflammatory response that is not predominant in tendinosis. The intervention with injectable is often done after a few months (3–6 months) of conservative physiotherapy during which the patient suffers considerably especially if the treatment is not working. In addition, alternative treatment such as immobilization may be deleterious than curative.

At present, few known regenerative injection-based therapies that have been used in supraspinatus and other tendinosis, in particularly lateral epicondylitis, are platelet rich plasma (PRP), autologous blood, and prolotherapy. Types of prolotherapy include dextrose, phenol-glycerine-glucose (P2G), and sodium morrhuate. Present data suggested that prolotherapy has a beneficial outcome when compared to the baseline status; however, more controlled clinical research studies are required to support these findings [12]. Therefore, the aim of this study is to study the role of dextrose prolotherapy and the ultrasound changes pre- and postinjection. Injections were given under ultrasound guidance into the area of tendinosis in the rotator cuff tendons of patients who are refractory to conservative medical treatment. The null hypothesis is prolotherapy injection do not provide faster relief in the patients' symptoms than the conventional therapy.

In this study, we evaluate the efficacy of dextrose prolotherapy injection for focal supraspinatus rotator cuff tendinosis via outcomes such as functional score, range of movement, and real-time ultrasound parameters. The premise of this study is that if a therapy is going to work, there will be a steep slope of improvement in the first few weeks of the therapy.

## 2. Materials and Methods

Twelve patients participated in this randomised controlled prospective study between August 2010 until November 2012. They were divided into two groups using random digit selection process, with odd number for the prolotherapy group (7 patients) and even number for the control group (5 patients). Inclusion criteria included duration of symptoms up to 6 months, supraspinatus tendinosis confirmed on ultrasound, and failure of functional score to improve more than 30% after 1 month of conventional treatment, which was physiotherapy and analgesics. Patients with mechanical impingement as cause of shoulder pain based on ultrasound dynamic testing for impingement were excluded from this study. Other exclusion criteria include autoimmune diseases, patients on anticoagulants, congenital or acquired platelet dysfunction abnormality/disorder, haemoglobin level less than 10 g/L and/or platelet count less than 100,000/*μ*L, corticosteroid or any shoulder injection within the past 6 weeks, and self-reported immunocompromised status.

Functional score using the Disability of Arm and Shoulder (DASH) Score and physical examination for range of shoulder movement were performed by the sports medicine physician at recruitment to study and at 12 weeks. A pain score of 1 to 5 is a subset of the DASH score. An improvement of pain is taken as reduction of 2 in the pain score scale. Meanwhile, ultrasound parameters assessed in this study were echogenicity area of tendinosis measured quantitatively in decibels (dB), area of tendinosis on cross section (mm^2^), length of partial tears (if present), presence of calcification, periostitis of adjacent greater tuberosity, Doppler flow within area of focal tendinosis, subacromial bursitis, and dynamic impingement.

All patients were examined using 18–5 MHz range linear probe on a Philips Epiq 5 ultrasound machine (Philips Healthcare, Best, Netherlands). All ultrasound measurement parameters were taken twice by two experienced musculoskeletal sonographers to assess for intraobserver and interobserver differences. The measurements on cross section were done at the same humeral head diameter for reproducibility. The echogenicity of the area of tendinosis and normal tendon was measured by placing a rectangular region of interest measuring 5 mm squared within obvious hypoechoic tendinosis and normal tendon in the same plane. Echogenicity was measured in decibels, and ratio of area of tendinosis to normal tendon was calculated.

**Figure 1:**
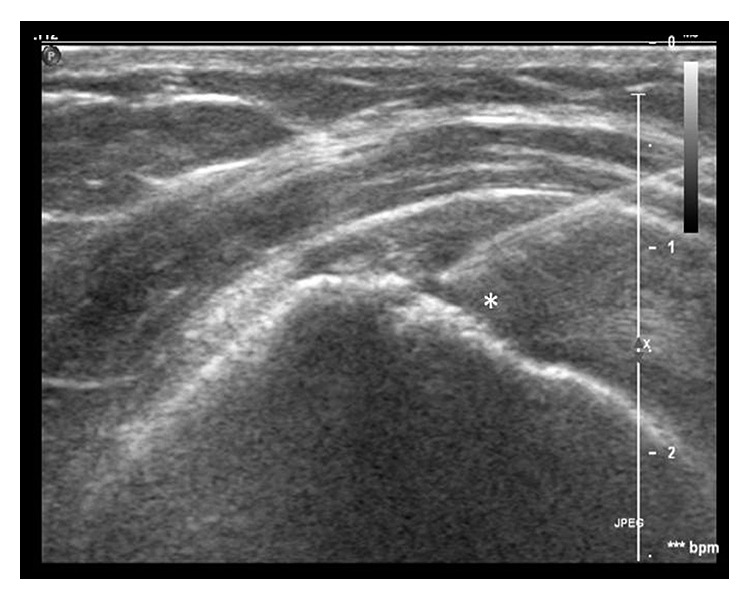
Longitudinal sonographic image obtained using a 5–17 MHz linear array transducer after insertion of a 21-gauge needle shows the tip of the needle located in the area of tendinosis with prolotherapy injected (^*∗*^).

Patients in the prolotherapy group were given an injection of 0.5–1.0 ml of mixture of 12.5% dextrose solution and 0.5% lignocaine in bacteriostatic water into area of painful tendinosis under ultrasound guidance and aseptic technique (Figure 1). Local anesthetic (lignocaine) was infiltrated along the intended tract prior to prolotherapy injection. The volume of the mixture injected depends on the degree of resistance during the injection and on spread of the solution within the tendon. Prior to the intratendinous injection, needling of the area of tendinosis was performed. Physiotherapy was resumed 2 weeks after injection. However, one patient in the control group had a progression of the injury to a full thickness tear at the end of 12 weeks and was not included in this study.

The data collected were nonparametric and analysed using Wilcoxon signed ranks test. Comparisons were made between the interventional group and the control group with regards to whether there was significant difference between both groups in improvement of ultrasound findings, functional scores, and range of motion using the Mann–Whitey *U* test and Fisher's exact test. All statistical analysis was performed using SPSS and *p* value < 0.05 taken to be of significance.

## 3. Results

The mean age in the prolotherapy group was 60 years old and 58 years old in the control group. The left shoulder (73.3%) was more affected than the right shoulder (36.7%). 82% of tendinosis in the supraspinatus were in the anterior aspect of the supraspinatus tendon. Seven (63.6%) patients had tendinosis at the articular side and 4 patients (36.4%) had tendinosis at the bursal side. All of the partial tears were located at the anterior aspect of the supraspinatus tendon. Apart from that, 3 of the prolotherapy patients (42.9%) have soft calcifications within the area of tendinosis.

In this study, there were ≥30% improvement of the DASH functional score at 12 weeks in 14.3% (1 patient) of patients in the prolotherapy group and 50% (2 patients) of patients in the control group. However, 50% of patients in the prolotherapy group have improvement between 21.2% and 28.3% of the functional score. The median difference in functional score from baseline to 12 weeks was 27.0% in the prolotherapy group and 17.9% in the control group. However, this difference was found to be not significant (*p*=0.364) (Table 1).

Even though there was an improvement in the mean pain score of patients in the prolotherapy group (43.5%) compared to the mean pain score of patients in the control group (25%), there was no significant difference in the pain score in both groups (*p*=0.247). On the contrary, the score for difficulty to sleep in the prolotherapy group had shown 34.6% reduction, whereas the control group had 18.2% increment at 12 weeks. There was a significant difference noted in the sleep improvement between the two groups (*p*=0.247) (Table 1).

**Figure 2:**
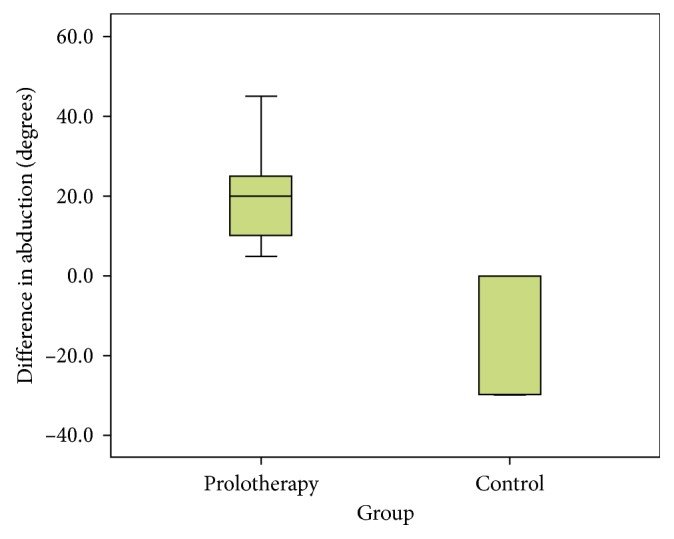
Boxplot comparing the difference in degree of abduction between the prolotherapy group and the control group at baseline and at 12 weeks (*p* value = 0.030).

In this study, we also found that the range of abduction of patients in the prolotherapy group increased with a mean of +20.0° while the mean range of patients in the control group decreased with a mean of −12.0°. There was significant improvement in shoulder abduction in prolotherapy patients compared to the control group using the Mann–Whitney *U* test (Figure 2). However, the other shoulder motions such as horizontal abduction, forward flexion, extension, internal rotation, external rotation, and horizontal adduction did not show any significant difference (*p* > 0.05).

**Figure 3:**
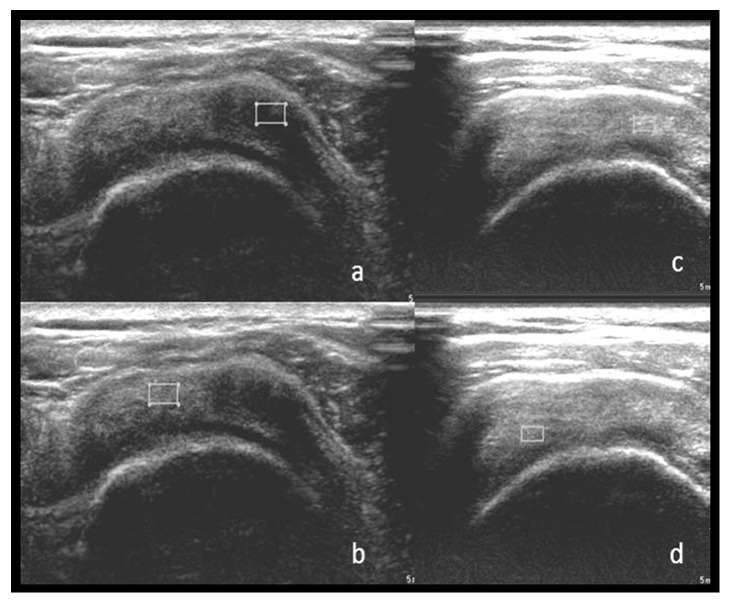
Echogenicity measurement of tendinosis (a) and normal tendon (b) at baseline, which was 5.56 dB and 19.50 dB, respectively, giving a ratio of 0.26. Echogenicity measurement of tendinosis (c) and normal tendon (d) at 12 weeks after injection, which was 20.07 dB and 28.97 dB, respectively, giving a ratio of 0.70, which showed an increase in ratio. Transverse sonographic image of the supraspinatus tendon at baseline (a, b) and at 12 weeks (c, d) at the same section showing almost similar humeral head diameter. The tendinosis measured with continuous trace (b) on cross section is almost not visible at 12 weeks (d) marked with 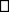.

There was median reduction in tear of −0.180 cm in the prolotherapy group and median increase in tear of +0.170 cm in tears in the control group at 12 weeks. However, the change in length of tear in both groups was not significant (*p*=1.00). Apart from that, there was no significant difference in improvement of the ratio echogenicity of tendinosis to normal tendon from baseline to 12 weeks between both groups using the Mann–Whitney *U* test(*p*=0.93). The median difference of area of tendinosis at baseline and at 12 weeks was 0.170 cm^2^ in the prolotherapy group and 0.310 cm^2^ in the control group. There was no significant difference in reduction of area of tendinosis at baseline and at 12 weeks between both groups using the Mann–Whitney *U* test (*p*=0.230). Conversely, there was a significant increase in the echogenicity of the combined tendinosis area from baseline to 12 weeks (*p*=0.009). The echogenicity and ratio measurement of tendinosis and normal tendon at baseline and at 12 weeks after injection are shown in Figure 3.

The other ultrasound parameters such as presence of calcification, Doppler flow, subacromial bursitis, periostitis, and impingement did not show significant difference between the prolotherapy group and the control group, as these features were only present in 3 patients. However, there was no significant correlation between these ultrasound parameters with functional and pain score (*p* > 0.05).

The ultrasound parameter measurements in each operator and between each operator using the Wilcoxon signed rank test showed no significant difference. The *p* value for median difference between the ultrasound parameters for readings in operator 1 was between 0.058 and 1.000, while for operator 2 it was between 0.075 and 0.937. The *p* value of interobserver median difference between operator 1 and operator 2 was between the ranges of 0.068 to 0.833.

The summary of the overall findings of the study is summarized in Table 2.

## 4. Discussion

The prevalence of tendinosis and tendon tears is mostly between the fifth to sixth decades of life with the size of the tear increasing with age [13, 14]. The mean age group of patients in this study was 58–60 years old which in keeping with tendinosis being part of a degenerative process. Most of the tendinosis and tears are located at the anterior aspect of the supraspinatus tendon and the full thickness tear recorded in the control group in this study was also located at the anterior aspect of the supraspinatus tendon. In a study on cadaveric shoulders by Hijioka et al., 60% of the shoulders showed degeneration at the subacromial surface, likely due to friction with the undersurface of the acromion [15]. A relatively hypovascular area within the supraspinatus tendon sited approximately 1 cm from the insertion on the greater tuberosity which predisposes to injury [16], in which diminished healing capacity in this hypovascular zone which increases with age [17].

Patients are suffering from chronic pain, loss of abduction, and loss of sleep for almost more than three months, and to ask the patients to wait for another three months before intervention will prolong their distress. Thus, the difference in this study is the early introduction of injectable after only a month of failed conservative therapy rather than the usual three to six months of failed conservative therapy in patients who showed less than 30% improvement in pain scores and functional scores after the one month of conservative physiotherapy. The findings of our study showed that earlier intervention could considerably help to alleviate the suffering in relation to shoulder abduction, and sleep scores should not be denied to patients just because the definition of chronic tendinopathy is 3 months of poor response to conservative therapy. Improving the shoulder function as early as possible should be the goal of any treating physician and not some guidelines, which do take this into account. In elite athletes and mechanical workers, the results of this study can be of great importance in terms of earlier return to play or work.

Again, the key findings of this study are that this ultrasound-guided intratendinous prolotherapy injection significantly improves patient's range of abduction and improves sleep and echogenicity within the area of tendinosis within 12 weeks duration, compared to conventional physiotherapy management. A retrospective study of prolotherapy for chronic shoulder pain showed overall disability reduction from 81% of the patients prior to prolotherapy to 20% after the injection. 87% of patients had 50% or greater relief of their shoulder pain, improvement in sleep, and less reliance on analgesics, and patients were less anxious and depressed with the relief of pain, with 97% of these patients feeling that the Hackett-Hemwall technique of dextrose prolotherapy injection did change their life for the better [18]. Even though there was no significant difference in DASH score seen in this study, on further breakdown of the DASH score, we found that there was significant improvement of sleep in the prolotherapy group. Patients will have better quality of sleep if they were able to lie on affected shoulder during sleep without being intermittently waking up in the middle of the night, and this will further improve their quality of life as they can focus during their daytime activities.

The action of supraspinatus muscle together with the deltoid muscle abducts the shoulder joint. During abduction, supraspinatus muscle pulls the head of the humerus inward toward the glenoid fossa [19]. In this study, patients of the prolotherapy group have a significant increase in the shoulder abduction (+20.0°). This is very important for patients to perform daily living activities; a simple action such as flicking on a light switch will be made possible with the improvement of the abduction and forward flexion. There was a similar type of joint range improvement seen in a study on prolotherapy injection in patients with knee osteoarthritis, in which patients had 40% decrease in pain after 12 months' dextrose prolotherapy injection and improvement of knee flexion of 14° [20]. However, the correlation between shoulder range of movements and ultrasound parameters was not significant, and this probably is due to our small sample size study population.

In regards to the sonography finding, there was an increase in the echogenicity of tendinosis area at 12 weeks of the study, which suggested that the changes had reformed to near-normal tendon that signify the tendon was at the remodeling phase of healing. During the remodeling phase, there was a decrease in the collagen and glycosaminoglycan synthesis, and the repaired tissue changed from cellular to fibrous tissue. The collagen fibres will align according to the direction they are stressed [18]. Similar to a study done using autologous blood injection for lateral epicondylitis, the median echogenicity of the tendon was significantly increased from 7 to 2 leading to near-normal-like tendon appearance using a semiquantitative score of 1–10, where 0 represents normal echogenic tendon and 10 represents diffuse hypoechoic change seen throughout the entire common tendon origin [18, 21]. Although these scores give a quantitative measurement, the echogenicity of the tendon is still subjectively assessed and will vary between operators. Zeisig et al. [22] and Connell et al. [23] reported that despite the presence of decreased structural defects on ultrasound, the improvement was not reliably correlated with clinical gains. For that reason, the measurement of echogenicity in our study was done objectively and reproducible between operators, as the measurement of echogenicity and its ratio were quantified in decibels.

As mentioned before, the other ultrasound parameters found was the presence of soft calcifications in the area of tendinosis in three of the prolotherapy patients, however, at the end of 12 weeks, the calcification in two of the patients was noted unchanged. Similar to a study done by Maxwell et al., looking at the intratendinous calcifications in chronic Achilles tendinosis, they reported that the calcification did not change following treatment in seven tendons [24]. On the contrary, one patient's calcific tendinosis in our study showed an improvement, likely due to the needling. Needling involves puncturing the calcium in the rotator cuff tendon up to 10–15 times to fragment it with 21 Gauge needle but there is concern of potential injury to the tendon caused by multiple punctures of the intratendinous calcifications with large-bore needles. However, there is no study yet published in the literature describing a less aggressive percutaneous technique. The calcific tendonitis processes are described into four stages: precalcific, calcific, resorptive, and postcalcific. During the resorptive stage when there is vascular invasion and migration of phagocytic cells and edema, sharp acute pain that limits shoulder movement occurs due to intratendinous pressure [25]. We found that no significant correlation was seen between calcification with function and pain in this study, which could be due to calcification being at different stages in our patients.

## 5. Conclusion

A modest clinical improvement was seen with the administration of dextrose prolotherapy in tendinopathy, and it was effective in the treatment of pain with joint movement limitation. Ultrasound-guided intratendinous prolotherapy injection significantly improves patient's range of abduction and improves sleep within 12 weeks compared to conventional physiotherapy management. It is advocated for patients who want faster improvement in shoulder functions, and especially important for elite athletes to return to sports.

## Figures and Tables

**Table 1:** Comparison of DASH, pain, and difficulty to sleep between the prolotherapy group (*n*=7) and the control group (*n*=4) at baseline and at 12 weeks.

	Mean		Post-pre- improvement (%)	*p* value (Fisher's exact test)
	Baseline	12 weeks		
*DASH score*				
Prolotherapy	60.14	43.89	27.0%	0.364
Control	56.86	46.68	17.9%	

*Pain score*				
Prolotherapy	3.29	1.86	43.5%	0.247
Control	3.20	2.40	25.0%	

*Difficulty to sleep score*				
Prolotherapy	3.29	2.15	34.6%	0.027^*∗*^
Control	2.20	2.60	−18.2%	

^*∗*^Level of significant set at *p* < 0.05.

**Table 2:** Summary of overall findings of this study.

Our study showed the following findings:
1. There was a 20 degree improvement in abduction ROM, which is statistically significant (*p*=0.03), but only modest in its magnitude.
2. A greater percentage of patients had a decrease in their DASH pain score of 2 or greater, compared to controls, but the comparison of the mean difference in DASH pain score between treatment and control did not reach statistical significance.
3. There was a statistically significant difference in sleep score improvement between intervention and control.
4. The prolotherapy group showed a statistically significant improvement in echogenicity within the area of tendinosis, which is an indication of healing.
